# Pharmacokinetic and exploratory exposure–response analysis of pertuzumab in patients with operable HER2-positive early breast cancer in the APHINITY study

**DOI:** 10.1007/s00280-019-03826-1

**Published:** 2019-04-11

**Authors:** Whitney P. Kirschbrown, Matts Kågedal, Bei Wang, Lars Lindbom, Adam Knott, Rachelle Mack, Sharareh Monemi, Ihsan Nijem, Sandhya Girish, Christie Freeman, Debora Fumagalli, Robin McConnell, Guy Jerusalem, Chris Twelves, José Baselga, Gunter von Minckwitz, José Bines, Amit Garg

**Affiliations:** 1 Genentech, 1 DNA Way, South San Francisco, CA 94080 USA; 2 qPharmetra, Hälsovägen 7, 141 57 Huddinge, Sweden; 3 Roche Products Limited, 6 Falcon Way, Shire Park, Welwyn Garden City, AL7 1TW UK; 4 Breast European Adjuvant Study Team (BrEAST) Data Center, Institut Jules Bordet, Boulevard de Waterloo 121 (7th Floor), 1000 Brussels, Belgium; 5 Breast International Group, Boulevard de Waterloo 76, 1000 Brussels, Belgium; 6 Frontier Science (Scotland), Grampian View, Kincraig, Inverness-Shire PH21 1NA UK; 7 International Breast Cancer Study Group, CHU Liège and Liège University, Domaine Universitaire du Sart Tilman, B35, 4000 Liège, Belgium; 8 University of Leeds and Leeds Teaching Hospitals Trust, St James’s University Hospital, Beckett Street, Leeds, LS9 7TF UK; 9 Executive Vice-President Research & Development Oncology, AstraZeneca, 950 Wind River Ln, Gaithersburg, MD 20878 USA; 10 German Breast Group, GBG Forschungs, Martin-Behaim-Str. 12, 63263 Neu-Isenburg, Germany; 11 Instituto Nacional de Câncer, Praça Cruz Vermelha, 23-Centro, Rio de Janeiro, 20230-130 Brazil

**Keywords:** Pharmacokinetics, Exposure–response, Drug–drug interactions, Pertuzumab

## Abstract

**Purpose:**

To characterize the pharmacokinetics (PK) of, and perform an exploratory exposure–response (E–R) analysis for, pertuzumab in patients with HER2-positive early breast cancer (EBC) within the APHINITY study (NCT01358877, BIG 4–11/BO25126/TOC4939G).

**Methods:**

A previously developed pertuzumab two-compartment linear population pharmacokinetic (popPK) model was subjected to external validation to examine appropriateness for describing pertuzumab concentrations from the APHINITY study. Pharmacokinetic drug–drug interactions (DDIs) between pertuzumab, trastuzumab, and chemotherapy were assessed by comparing observed serum or plasma *C*_max_, *C*_min_, and AUC_last_ geometric mean ratios with 90% CIs. Predictions of pertuzumab *C*_max,ss_, *C*_min,ss_, and AUC_ss_ were derived from individual parameter estimates and used in an exploratory E–R analysis.

**Results:**

Using data from 72 patients, based on goodness-of-fit, the popPK model was deemed appropriate for predictions of individual exposures for subsequent comparisons to historical data, assessment of DDIs, and E–R analyses. No evidence of DDIs for pertuzumab on trastuzumab, trastuzumab on pertuzumab, or pertuzumab on chemotherapy PK was observed. Analyses of differences in exposure between patients with and without invasive disease-free survival events did not indicate improved efficacy with increased exposure. Overall Grade ≥ 3 diarrhea prevalence was higher with pertuzumab versus placebo, but was not greater with increasing pertuzumab exposure. No apparent E–R relationship was suggested with respect to other grade ≥ 3 AEs.

**Conclusion:**

Overall, the limited available data from this exploratory study suggest that no dose adjustments are needed for pertuzumab when administered in combination with trastuzumab and an EBC chemotherapy regimen.

**Electronic supplementary material:**

The online version of this article (10.1007/s00280-019-03826-1) contains supplementary material, which is available to authorized users.

## Introduction

Pertuzumab (PERJETA^®^, F. Hoffmann-La Roche Ltd, Basel, Switzerland) is a recombinant, humanized immunoglobulin G1κ monoclonal antibody that targets human epidermal growth factor receptor 2 (HER2), a transmembrane glycoprotein with intrinsic tyrosine kinase activity [1]. By binding to extracellular subdomain 2, pertuzumab prevents dimerization of HER2 with other HER family receptors [2, 3]. As a result, pertuzumab inhibits two major ligand-initiated intracellular signaling pathways, mitogen-activated protein kinase and phosphoinositide 3-kinase, thereby inducing cell growth arrest and apoptosis [4].

Pertuzumab and trastuzumab (Herceptin^®^, F. Hoffmann-La Roche Ltd) bind to different epitopes on the HER2 receptor and have distinct mechanisms for disrupting HER2 signaling. Due to their complementary modes of action, the combination of these two anti-HER2 antibodies provides more comprehensive HER2 pathway blockade than single agents [5, 6]. Pertuzumab was first approved for use in combination with trastuzumab and docetaxel in patients with HER2-positive metastatic breast cancer (MBC), based on the pivotal phase III CLEOPATRA study (NCT00567190), which showed significant improvement in progression-free survival with the combination as compared with placebo plus trastuzumab and docetaxel [7].

Pertuzumab is also licensed as neoadjuvant treatment for patients with HER2-positive early breast cancer (EBC); approval was supported by two phase II studies, NeoSphere (NCT00545688) and TRYPHAENA (NCT00976989), in which the addition of pertuzumab to trastuzumab and chemotherapy significantly improved pathologic complete response rate (pCR), without increasing cardiac toxicities [8, 9].

The clinical pharmacokinetics (PK) of pertuzumab were first described in 481 patients with a variety of solid tumors from 11 phase I/II studies and CLEOPATRA [10]. A two-compartment linear model with first-order elimination was used to characterize pertuzumab PK in the 2–25 mg/kg dose range, a range which includes the approved fixed-dose regimen of an 840 mg loading dose followed by a 420 mg maintenance dose, administered intravenously on an every-3-week (q3w) schedule [10]. In the final model of this analysis, covariates explained 21.6% of the between-subject variability for clearance (CL) and 35.0% of the between-subject variability for volume of central compartment (*V*_c_), with lean body weight (LBW) and serum albumin (ALBU) being identified as statistically significant covariates of pertuzumab PK [10]. LBW impacted pertuzumab PK: increased LBW correlated with increasing CL, *V*_c_, and volume of peripheral compartment (*V*_p_) [10]. Sensitivity analyses demonstrated that the magnitude of its effect on the pertuzumab exposure measures minimum concentration at steady state (*C*_min,ss_), maximum concentration at steady state (*C*_max,ss_), and area under the concentration–time curve at steady state (AUC_ss_) was small relative to the overall between-subject variability in the population [10]. The authors concluded that, because of this, dose adjustment for LBW is not warranted [10].

The maximum tolerated dose for pertuzumab was not reached in clinical studies; therefore, the selected dose for CLEOPATRA and NeoSphere was based on achievement of a trough concentration at steady state (*C*_trough,ss_) of ≥ 20 μg/mL in 90% of patients (i.e., clinical target concentration) [11]. Non-clinical xenograft dose–response studies showed maximal suppression of tumor growth when the *C*_trough_ was maintained above this threshold [12]. Over 90% of patients achieved the target serum pertuzumab concentration in the NeoSphere study, and the exposure–response analysis suggested that there was no association between pCR rate and pertuzumab concentrations within the observed concentration range of 20–100 μg/mL [11]. This analysis further supported the appropriateness of the fixed, non-weight-based pertuzumab dose of 840 mg followed by 420 mg q3w in the neoadjuvant treatment of patients with early breast cancer [11].

More recently, pertuzumab was approved for the adjuvant treatment of patients with HER2-positive EBC based on the APHINITY study (NCT01358877, BIG 4–11/BO25126/TOC4939G). APHINITY was a prospective, randomized, multicenter, multinational, double-blind, placebo-controlled, phase III study that compared intravenous pertuzumab (18 cycles), trastuzumab (18 cycles), and chemotherapy (3–4 cycles of anthracycline-containing chemotherapy followed by 3–4 cycles of taxane-containing chemotherapy or 6 cycles of docetaxel plus carboplatin) with placebo, trastuzumab, and chemotherapy as adjuvant therapy in patients with operable HER2-positive early breast cancer (EBC) [13]. APHINITY met its primary objective, showing significantly improved rates of invasive disease-free survival (IDFS) with the addition of pertuzumab to trastuzumab and chemotherapy. A disease recurrence event occurred in 7.1% of patients treated with pertuzumab and in 8.7% of patients who received placebo (a difference of 1.6%; hazard ratio 0.81; 95% confidence interval [CI] 0.66–1.00; *p* = 0.045). In terms of safety, diarrhea (a common adverse event with pertuzumab) of Grade 3 or higher occurred more frequently with pertuzumab than with placebo, and was mostly experienced during chemotherapy [13].

Data described herein were collected as part of an optional APHINITY Global PK sub-study; designed to characterize pertuzumab steady-state pharmacokinetics in patients with HER2-positive EBC and to support the current pertuzumab dosing regimen in this population. The key objectives for this PK analysis were to: (1) characterize the steady-state PK of pertuzumab in patients with HER2-positive EBC; (2) characterize the potential PK drug–drug interactions (DDIs) between the therapeutic proteins trastuzumab and pertuzumab, and between pertuzumab and paclitaxel and carboplatin. In addition, three exploratory objectives addressed by this PK analysis were to: (1) compare the steady-state concentrations of pertuzumab, when administered as adjuvant treatment to patients with EBC, with data obtained previously in women with MBC (CLEOPATRA); (2) compare the steady-state concentrations of trastuzumab when administered as adjuvant treatment to patients with EBC, with data obtained previously in women with EBC receiving adjuvant treatment (HERA [NCT00045032]); (3) perform an exploratory analysis of exposure–response relationships, including IDFS and diarrhea as the key efficacy and safety endpoints, respectively.

## Materials and methods

### APHINITY Global PK sub-study design

The design of the APHINITY study has been reported previously [13]. APHINITY included an optional Global PK sub-study with a separate protocol from the main trial.

### PK sampling

PK samples were collected only in the PK sub-study. A sparse PK sampling approach was adopted. The PK sampling times for pertuzumab and trastuzumab were pre- and post-dose on cycles 1, 10, and 15 (and Cycle 2 pre-dose). PK sampling times for chemotherapy included 3, 5, and 24 h post-dose for paclitaxel, 3 and 5 h post-dose for the metabolite 6-alpha-hydroxy paclitaxel, and 1, 2, 4, and 5 h post-dose for carboplatin.

### Bioanalytical methods

Validated assays were used to measure pertuzumab, trastuzumab, paclitaxel and 6-alpha-hydroxy paclitaxel, and carboplatin from blood samples.

The serum concentrations of pertuzumab were determined by an enzyme-linked immunosorbent assay described previously [14]. The assay used a monoclonal anti-idiotype antibody against pertuzumab to capture pertuzumab from serum samples. Bound pertuzumab was detected with a biotinylated monoclonal antibody (10C4; Antibody Engineering, Genentech, Inc., South San Francisco, CA, USA) against a Genentech, Inc. immunoglobulin G framework and horseradish peroxidase-Avidin D conjugate. A peroxidase substrate (tetramethyl benzidine) was used for color development to quantify serum pertuzumab against a standard curve. The lower limit of quantification in human serum was 150 ng/mL with a standard curve reporting range of 150–4000 ng/mL (limit of detection was 62.5 ng/mL). The inter-assay accuracy (percentage difference) ranged from − 8.75 to 3.84% while the inter-assay precision (percentage coefficient of variation) ranged from 3.89 to 15.3%. The presence of trastuzumab did not interfere with the accurate quantification of pertuzumab in this assay.

Trastuzumab serum concentrations were determined by a validated high-performance liquid chromatography with tandem mass spectrometry (LC–MS/MS) detection described previously [15]. An affinity capture approach using streptavidin magnetic beads coupled with biotinylated recombinant human HER2 extracellular domain was used to enrich trastuzumab from human serum. The bound trastuzumab protein was subjected to ‘on-bead’ proteolysis with trypsin, following standard protein denaturation, reduction, and alkylation processing steps. Prior to digestion completion, working internal standard solution was added. The characteristic peptide fragments produced by this procedure were then quantified as surrogates of the total antibody concentration originating from trastuzumab by LC–MS/MS (i.e., multiple reaction monitoring [MRM]). The lower limit of quantification in human serum was 100 ng/mL with a standard curve reporting range of 100 ng/mL to 2500 ng/mL. The inter-assay accuracy (percentage difference) ranged from − 8.08 to − 1.47%, while the inter-assay precision (percentage coefficient of variation) ranged from 3.07 to 8.44%. The presence of pertuzumab did not interfere with the accurate quantification of trastuzumab in this assay.

Plasma concentrations of paclitaxel and its metabolite 6-alpha-hydroxy paclitaxel were determined by a validated liquid chromatography tandem mass spectrometry method. An aliquot of 50 μL of human plasma (K_2_EDTA) sample containing paclitaxel and 6-alpha-hydroxy paclitaxel was extracted using supported-liquid extraction. The API 5000 Triple Quad™ (Applied Biosystems, Foster City, CA, USA) was operated in MRM mode under optimized conditions for the detection of paclitaxel and 6-alpha-hydroxy paclitaxel positive ions formed by electrospray ionization. Paclitaxel-d_5_ was used as an internal standard. Paclitaxel concentrations were calculated with the use of a standard curve with a 1/*x*^2^ linear regression over a concentration range of 2.00–2500 ng/mL. Concentrations of 6-alpha-hydroxy paclitaxel were calculated using a separate standard curve with a 1/*x*^2^ linear regression over the same concentration range of 2.00–2500 ng/mL. The inter-assay relative standard deviation ranged from 1.5 to 9.6% for paclitaxel and from 2.2 to 8.9% for 6-alpha-hydroxy paclitaxel. The inter-assay accuracy ranged from 86.0 to 96.6% of nominal for paclitaxel and from 96.5 to 103.2% of nominal for 6-alpha-hydroxy paclitaxel. Stability of paclitaxel and 6-alpha-hydroxy paclitaxel was established in human plasma for 449 days at − 20 °C and 1280 days at – 70 °C.

Carboplatin plasma concentrations were determined by a validated inductively coupled plasma tandem mass spectrometry method. Human plasma (K_2_EDTA) samples (50 μL) containing carboplatin were analyzed on a Perkin-Elmer ELAN DRC II mass spectrometer optimized for the detection of platinum from carboplatin. Terbium was used as an internal standard. Platinum concentrations were calculated with the use of a standard curve with a 1/*x*^2^ linear regression over a concentration range of 2.00–1000 ng/mL. The inter-assay relative standard deviation ranged from 0.68 to 4.06%, while the inter-assay accuracy ranged from 97.6 to 100.8% of nominal. Stability of platinum was established in human plasma for 195 days at − 20 °C and − 70 °C.

### Data handling

Patients were defined as evaluable for pharmacokinetic (PK) analysis if they had at least one documented pertuzumab administration and a corresponding post-dose pertuzumab PK sample collection. Records were excluded if the time of drug administration or sample collection was missing. No imputation of PK values was performed. Observations with missing PK or time values, or those below the minimum quantifiable concentration, were omitted from the analysis.

Outliers were identified by visual inspection of each individual’s concentration versus time profile. Typically, a data point was deemed an outlier if a trough concentration was greater than the peak concentration, or if the absolute residual variability was five times larger than the expected residual standard deviation.

### PK analysis

Pertuzumab concentrations from the APHINITY Global PK sub-study were compared with predictions based on a previously developed pertuzumab population PK model [10]. This model was built on data collected from patients with solid tumors, including MBC, during five phase I/Ib studies, six phase II studies, and one pivotal phase III study [10]. Most of the data ( > 95%) used for population PK development were based on pertuzumab without concomitant trastuzumab treatment; seven of the 12 studies included investigated pertuzumab as a monotherapy. In the previously developed model, pertuzumab PK were described by a two-compartment linear model with a CL, central volume of distribution, and terminal elimination half-life of 0.235 L/day, 3.11 L, and 18 days, respectively. The covariates identified as significantly influencing pertuzumab CL were baseline serum albumin and LBW, with 15.5% and 4.1% of the between-subject variability in CL explained by serum albumin and LBW, respectively.

Comparisons of pertuzumab concentrations from the APHINITY Global PK sub-study to the previously developed model predictions were performed using NONMEM version 7.3 software (ICON Development Solutions, Ellicott City, MD, USA). Post-processing of NONMEM analysis results was carried out in R version 3.2.2 (R Development Core Team, R: A language and environment for statistical computing. R Foundation for Statistical Computing, Vienna, Austria; ISBN 3-900051-07-0; URL https://www.R-project.org/). Individual PK parameters were estimated using first-order conditional estimation with interaction.

To evaluate the agreement of the observed PK data in the APHINITY Global PK sub-study with the historical PK data based on the population PK model, a visual predictive check was performed. Pertuzumab serum concentrations for 10 000 subjects were simulated using LBW resampled from the observed LBW in the pertuzumab arm as well as nominal dose times and amounts for each patient. Albumin levels were not measured in APHINITY. Therefore, the median observed baseline albumin level of 4.3 g/dL (range 3.3–5.7 g/dL, *N* = 258) in HannaH (NCT00950300), a study of subcutaneous or intravenous trastuzumab for EBC [16], was added as the value for those in the APHINITY Global PK sub-study. In the NeoSphere study, the median observed baseline albumin level was 4.4 g/dL (range 3.1–5.3 g/dL, *N* = 180), indicating that the selected median baseline albumin value of 4.3 g/dL is appropriate for an EBC patient population and can be used to describe pertuzumab PK in the APHINITY study. Median predicted pertuzumab concentrations and a 95% prediction interval were compared with the observed data.

For the purpose of the exploratory exposure–response analysis, individually predicted pertuzumab serum concentrations based on each patient’s observed serum concentrations and covariates were obtained. The predictions were derived by fixing the parameters in the structural and variance model to the parameter estimates in the historical validated population PK model and generating the individual empirical Bayes estimates with NONMEM by setting MAXEVAL = 0. Individual exposure estimates (AUC, *C*_min_, and *C*_max_) were subsequently obtained for use in the exposure–response analysis (detailed below). Diagnostic plots of observed data versus population prediction and individual prediction were examined for adequate fit. Plots of conditional weighted residual versus population prediction and versus time (after first and last doses) were inspected for evidence of systematic lack of fit, and to confirm the absence of bias in the error distributions.

### DDIs

The DDI analysis was carried out using R version 3.2.2.

The potential effect of pertuzumab on the steady-state PK of trastuzumab was assessed by comparing the arithmetic means of serum trastuzumab concentrations at pre-dose (*C*_min,ss_) and post-infusion (*C*_max,ss_) in cycles 10 and 15 in the pertuzumab and placebo arms. In addition, the 90% CIs in the ratio of the geometric means (calculated by standard methods) were constructed. Similarly, the potential effect of pertuzumab on the PK of paclitaxel (and 6-alpha-hydroxy paclitaxel) and carboplatin was assessed by comparing the arithmetic means of *C*_max_ and area under the concentration–time curve over all concentration measurements (AUC_last_) in Cycle 1 in the pertuzumab and placebo arms.

For paclitaxel and carboplatin, collection of multiple blood samples on Cycle 1 Day 1 allowed characterization of the post-infusion concentration–time curves using noncompartmental methods. *C*_max_ was defined as the maximum observed concentration and AUC_last_ was calculated using the linear trapezoidal rule and nominal observation times. The 90% CIs in the ratio of the geometric means were also constructed. All observations for 6-alpha-hydroxy paclitaxel at 24 h post-dose were reported as below the quantification limit. Thus, AUC_last_ was not calculated.

The potential effect of trastuzumab on the PK of pertuzumab was assessed by comparing pertuzumab *C*_max_ and *C*_min_ observed in the APHINITY Global PK sub-study with the predictions based on the population PK model. An adequate prediction of the observed PK by the historical model would suggest that there was no impact of trastuzumab on the PK of pertuzumab.

### Exploratory exposure–response analysis

To generate individual pertuzumab exposure for patients in the pertuzumab arm, a simulation dataset was constructed based on estimated individual parameters for the population PK model, corresponding LBW and median albumin incorporated as described previously. For all patients with at least one available valid post-treatment concentration measurement of pertuzumab, predictions of *C*_max,ss_, *C*_min,ss_, and AUC at steady state (AUC_ss_) were derived. The simulation dataset consisted of a loading dose and three maintenance doses. The loading dose and infusion duration were taken from the NONMEM dataset for each patient in the pertuzumab arm; thereafter, three doses of 420 mg pertuzumab were administered using a 30-min infusion with 3 weeks’ dosing interval. Pertuzumab approximate steady state is achieved following the first maintenance dose, and therefore three maintenance doses were selected to ensure steady state across patients. Individual predictions of concentration at the end of infusion (*C*_max,ss_) and before next dose (*C*_min,ss_) were generated following the third maintenance dose for all patients. AUC_ss_ was calculated by dividing the maintenance dose by individual clearance values.

The efficacy endpoint in the exposure–efficacy analysis was the primary study endpoint, IDFS [13]. IDFS is the time from randomization to recurrence of ipsilateral invasive breast tumor, recurrence of ipsilateral locoregional invasive disease, a distant disease recurrence, contralateral invasive breast cancer, or death from any cause. Patients who had not had an event at the time of data analysis were censored at the date they were last known to be event-free. The primary exposure metrics used in the exposure–efficacy analysis were individual predicted *C*_min,ss_ and AUC_ss_. Box plots were created to compare exposure (*C*_min,ss_ and AUC_ss_) in the subset of patients with PK data who had an IDFS event (*N* = 4) versus the patients with PK data who did not (*N* = 31).

Adverse events (AEs) were considered for the analysis if there was a difference of ≥ 5% in the incidence of Grade ≥ 3 AEs between the pertuzumab and placebo arms in the primary analysis [13]. Furthermore, to be considered for the analysis, pertuzumab PK data were required for ≥ 5 patients experiencing the AE; “any grade ≥ 3 AE” (incidence 64.2% in the pertuzumab arm and 57.3% in the placebo arm in the overall population [13]) and Grade ≥ 3 diarrhea (incidence 9.8% in the pertuzumab arm and 3.7% in the placebo arm in the overall population [13]) met these criteria. These safety endpoints were assessed as binary variables. Results were deemed exploratory and did not reflect formal statistical hypothesis testing.

## Results

### Patients and samples

Seventy-two patients consented to the optional APHINITY PK Global sub-study. Of these, 38 patients received pertuzumab, and 35 contributed at least one pertuzumab PK sample. The total number of pertuzumab PK observations was 163. In addition, 36 patients in the pertuzumab arm and 34 in the placebo arm contributed at least one trastuzumab PK sample. The total number of trastuzumab PK observations was 339 (179 from the pertuzumab arm and 160 from the placebo arm).

Patient demographics are summarized in Online Resource 1. In the APHINITY Global PK sub-study, all 38 patients who received pertuzumab were female, with a median age of 51.5 years and a median LBW of 44.7 kg. These demographic characteristics, as well as nodal status and hormone receptor status, were comparable between the APHINITY Global PK sub-study and APHINITY intention-to-treat populations [13].

### Pertuzumab PK

**Fig. 1:**
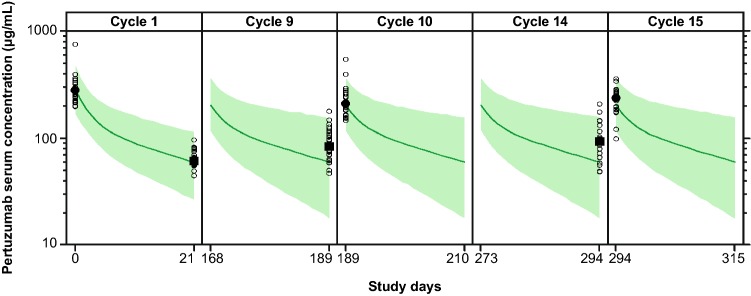
Visual predictive check of pertuzumab concentrations. The open circles represent observed serum concentrations. The filled circles represent median observed *C*_max_. The filled squares represent median observed *C*_min_. The solid green line represents predicted median serum concentration. The shaded area represents 95% prediction interval*. C*_max_ is maximum serum concentration. *C*_min_ is minimum serum concentration

**Fig. 2:**
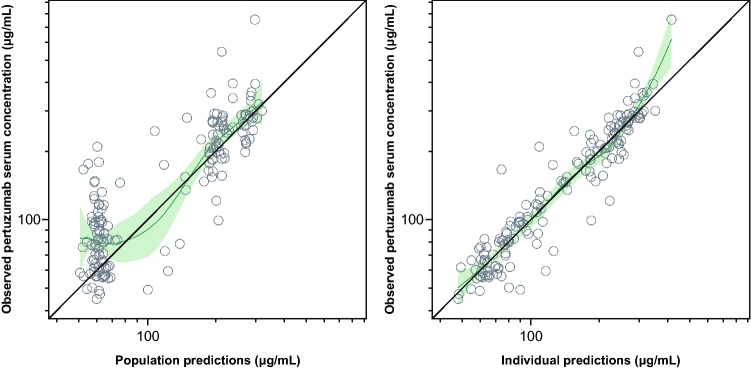
Observed versus model-predicted pertuzumab serum concentrations. The solid black line represents line of identity. The solid green line and shaded green area are Gaussian Loess smooth with 95% confidence interval. The left-panel circles are individual observations/population predictions. The right-panel circles are individual observations/individual predictions

The visual predictive check of the population PK model for the Global PK sub-study data is shown in Fig. 1. Overall, the population PK model predicted the serum concentrations reasonably well across cycles. *C*_min_ appeared to increase in later cycles, although the majority of the observed pertuzumab concentrations were within the 95% prediction interval. Graphical assessment of the match between observed and model-predicted pertuzumab concentrations (Fig. 2), as well as of conditional weighted residuals (Online Resource 2), suggested that the previous model could predict the observed data reasonably well. However, the observed trough concentrations at later cycles were slightly higher than predicted by the model. Based on goodness of fit, the model was deemed appropriate for predictions of individual exposures for subsequent comparisons with historical data, assessment of potential DDIs, and exposure–response analyses.

### DDIs

**Fig. 3:**
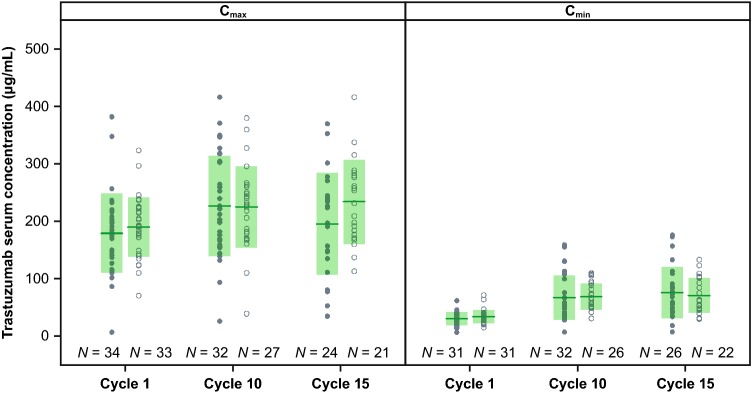
Trastuzumab *C*_max_ and *C*_min_ with or without pertuzumab. The closed circles represent trastuzumab in the treatment arm (pertuzumab, trastuzumab, and chemotherapy). The open circles represent trastuzumab in the control arm (placebo, trastuzumab, and chemotherapy). The solid green line represents arithmetic mean for each timepoint and treatment arm. The shaded area is arithmetic mean ± 1 standard deviation. *C*_max_ is maximum serum concentration. *C*_min_ is minimum serum concentration

The *C*_min_ and *C*_max_ of trastuzumab with pertuzumab or placebo across cycles are shown and summarized in Fig. 3 and Online Resource 3, respectively. Trastuzumab geometric mean ratios were approximately 0.8–1.0 and the 90% CIs all overlapped 1.0, indicating no impact of pertuzumab on trastuzumab serum *C*_min_ or *C*_max_ when administered in combination with an EBC chemotherapy regimen. There was also no evidence to suggest an impact of pertuzumab (in combination with trastuzumab) on PK of paclitaxel (*C*_max_ and AUC), 6-alpha-hydroxy paclitaxel (*C*_max_ only, as the observations at 24 h after dose were below the quantification limit) or carboplatin (*C*_max_ and AUC). The data are shown in Online Resources 4, 5, and 6, respectively. It should be noted that carboplatin PK data were derived from only six patients in the pertuzumab arm and 12 patients in the placebo arm. An adequate prediction of observed pertuzumab PK in APHINITY by the historical model as described above suggested that there was no impact of trastuzumab on the PK of pertuzumab.

### Pertuzumab and trastuzumab exposure in EBC and MBC studies

Serum pertuzumab *C*_min_ and *C*_max_ data in the APHINITY Global PK sub-study (cycles 1, 10, and 15) were comparable to PK data from the CLEOPATRA study in MBC (cycles 3, 9, and 15) as shown in Table 1.

**Table 1:** Summary of *C*_min_ and *C*_max_ of pertuzumab in the presence of trastuzumab with and without chemotherapy (upper panel), and trastuzumab (lower panel) in patients with early breast cancer or metastatic breast cancer

Pertuzumab									
APHINITY (EBC)					CLEOPATRA (MBC)^a^				
Cycle	*n*	*C*_min_ (µg/mL)	*n*	*C*_max_ (µg/mL)	Cycle	*n*	*C*_min_ (µg/mL)	*n*	*C*_max_ (µg/mL)
1	30	65.9 (12)	30	291.2 (99)	3	18	63.4 (48)	18	183.4 (34)
10	30	91.0 (31)	28	229.8 (83)	9	16	75.5 (22)	14	196.3 (66)
15	24	98.4 (40)	21	232.8 (65)	15	11	94.1 (31)	9	221.1 (32)

Trastuzumab									
APHINITY (EBC)					HERA (EBC)^a^				
Cycle	*n*	*C*_min_ (µg/mL)	*n*	*C*_max_ (µg/mL)	Cycle	*n*	*C*_min_ (µg/mL)	*n*	*C*_max_ (µg/mL)
10	58	67.7 (32)	59	225.5 (80)	10	3	66.0 (39)	3	203 (7)
15	48	73.6 (38)	45	213.0 (83)	12	15	72.3 (46)	15	237 (12)

Arithmetic means (SD)

*C*_*max*_ is maximum serum concentration, *C*_*min*_ is minimum serum concentration, *EBC* is early breast cancer, *MBC* is metastatic breast cancer, *SD* is standard deviation

^a^Roche data on file; cycles 10 and 15 of APHINITY were chemotherapy-free

Serum trastuzumab *C*_min_ and *C*_max_ data at later cycles in the APHINITY Global PK sub-study (cycles 10 and 15) were comparable to observed trastuzumab PK data from the experimental arm of the HERA study in EBC (cycles 10 and 12), as demonstrated in Table 1. It should be noted that the observed trastuzumab data from APHINITY were pooled from pertuzumab and placebo arms, after confirming no PK DDI between pertuzumab and trastuzumab in the APHINITY Global PK sub-study.

### Exposure–efficacy analysis

**Fig. 4:**
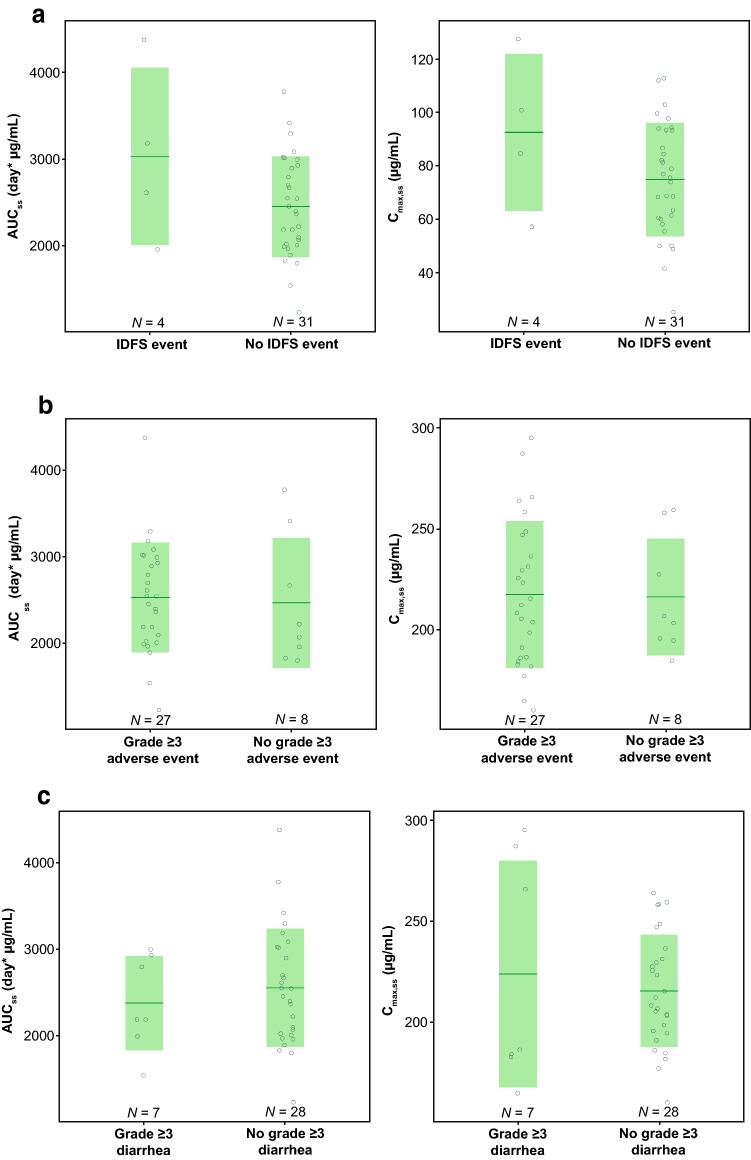
The relationships between pertuzumab exposure and efficacy or safety. Pertuzumab AUC_ss_, *C*_min,ss_, or *C*_max,ss_ for patients in the pertuzumab arm with or without invasive disease-free survival (IDFS) event (**a**), any grade ≥ 3 adverse event (AE) (**b**) or any grade ≥ 3 diarrhea (**c**). The solid green line represents arithmetic mean for each group. The shaded area represents arithmetic mean ± 1 standard deviation. *AUC*_*ss*_ is area under the concentration–time curve at steady state. *C*_max*,ss*_ is maximum serum concentration at steady state. *C*_min*,ss*_ is minimum serum concentration at steady state

Thirty-five patients in the pertuzumab arm with predicted AUC_ss_ and *C*_min,ss_ were included in the exploratory exposure–efficacy analysis. There was no indication of improved efficacy, as defined by IDFS, with higher exposure to pertuzumab (AUC_ss_ and *C*_min,ss_; Fig. 4a). However, it should be noted that only four IDFS events occurred in the PK sub-study population, so there were limited data available for the analysis.

### Exposure–safety analysis

In the safety dataset, 35 patients treated with pertuzumab had PK exposure predictions, and 34 patients in the placebo arm were used as a reference group to compare the potential exposure–response of pertuzumab. Table 2 summarizes the occurrence of Grade ≥ 3 AEs for patients by treatment arm, as well as the patients treated with pertuzumab with exposure (AUC_ss_ or *C*_max,ss_) below or above median. The possible relationships between AUC_ss_ or *C*_max,ss_ and occurrence of any grade ≥ 3 AEs are shown in Fig. 4b; the incidence of any grade ≥ 3 AEs was similar in the pertuzumab and placebo arms, and the incidence of Grade ≥ 3 AEs was similar in the high and low pertuzumab exposure groups (approximately 10% difference but a difference of only one patient in the two groups).

**Table 2:** Grade ≥ 3 AEs by PK group

Group	*n*	Any grade ≥ 3 AE^a^	Grade ≥ 3 diarrhea
Overall			
Placebo + trastuzumab + chemotherapy	34	25 (73.5)	2 (5.9)
Pertuzumab + trastuzumab + chemotherapy	35	27 (77.1)	7 (20.0)
AUC_ss_			
Pertuzumab below the median	18	13 (72.2)	4 (22.2)
Pertuzumab above the median	17	14 (82.4)	3 (17.6)
*C*_max,ss_			
Pertuzumab below the median	18	13 (72.2)	4 (22.2)
Pertuzumab above the median	17	14 (82.4)	3 (17.6)

Data are patients, *n* or patients, *n* (%)

*AE* is adverse event, *AUC* is area under the concentration–time curve, *C*_*max*_ is maximum serum concentration, *PK* is pharmacokinetic

^a^The most common Grade ≥ 3 AEs were neutropenia, febrile neutropenia, neutrophil count decreased, diarrhea, and anemia [10]

The incidence of Grade ≥ 3 diarrhea was more frequent in pertuzumab-treated patients than in placebo-treated patients, but the occurrence of Grade ≥ 3 diarrhea did not increase with increasing pertuzumab exposure (Fig. 4c). It should be noted that grouping of patients based on AUC_ss_ or *C*_max,ss_ was identical, i.e., all patients with AUC_ss_ above the median also had a *C*_max,ss_ above the median. Hence, results were identical for *C*_max,ss_ and AUC_ss_.

## Discussion

The previously developed population PK model for pertuzumab, built on a large database of patients with MBC and other solid tumors [10], was used in the analyses of data collected from the APHINITY Global PK sub-study to characterize the PK of pertuzumab at steady state in the APHINITY patient population (EBC) and the potential interactions between pertuzumab and trastuzumab and chemotherapy. In addition, the steady-state concentrations of pertuzumab and trastuzumab were compared with historical data in patients with EBC or MBC, and the exposure–response relationships were also explored.

The previously developed and validated population PK model adequately predicted pertuzumab serum concentrations across cycles in APHINITY. Although *C*_min_ appeared to increase in later cycles, an increase of similar magnitude in pertuzumab *C*_min_ over time was also observed in CLEOPATRA and may be indicative of time-dependent PK or treatment effect [14]. A definitive conclusion regarding time-dependency in pertuzumab PK cannot be made due to the small sample size in APHINITY. Also, it should be noted that clinical efficacy is expected if the target *C*_trough_ for pertuzumab is maintained at or above 20 μg/mL, and the increase in *C*_min_ had no apparent impact on safety [11]. While the observed *C*_min_ at later cycles tended to be slightly higher than predicted by the model, there was a large overlap and overall the data suggested no clinically meaningful difference among patients with EBC and other tumor types, including MBC (CLEOPATRA). This was further supported by the comparable observed PK of pertuzumab in the APHINITY and CLEOPATRA studies. Given that a population with EBC treated in the adjuvant setting has had surgical removal of tumor, when PK is found to be comparable to MBC and other solid tumor types, it suggests that tumor burden has no apparent impact on pertuzumab PK. Overall, the data demonstrated that this population PK model can be used for predictions of pertuzumab exposure in patients with HER2-positive EBC, in addition to other solid tumors included when the original model was developed.

Although most of the data used to build the population PK model were based on pertuzumab without concomitant trastuzumab, *C*_min_ and *C*_max_ of pertuzumab from patients in the APHINITY Global PK sub-study were adequately described by the model. In addition, the general agreement between observed pertuzumab concentrations and the values predicted by the population PK model suggested that trastuzumab had no clinically significant impact on the PK of pertuzumab in APHINITY.

Observed serum pertuzumab *C*_min_ and *C*_max_ data in APHINITY were comparable to pertuzumab *C*_min_ and *C*_max_ from the CLEOPATRA study in MBC at similar cycles. Additionally, serum trastuzumab *C*_min_ and *C*_max_ data in APHINITY were comparable to observed trastuzumab PK data from the experimental arm of the HERA study in EBC at similar cycles. These comparisons showed that pertuzumab PK data were comparable across different indications and chemotherapy combination partners and therefore indicates no impact of disease and chemotherapy on pertuzumab PK.

DDIs were not expected in this study, based on prior data [11, 14] and the distinct clearance mechanisms between monoclonal antibodies and the cytotoxic agents evaluated [17–19]. As expected, PK parameters *C*_max_ and AUC_last_ for paclitaxel and carboplatin were found to be similar in both treatment arms, indicating no impact of pertuzumab on the PK of these chemotherapeutic agents. The 90% CIs of the ratios of the PK parameters were wide and were most probably affected by the variability in PK parameters and the low statistical power associated with the small sample sizes between the treatment groups.

Similarly, no difference in trastuzumab PK parameters were observed in the presence of pertuzumab. PK exposure ratios (pertuzumab arm vs. placebo arm) were close to 1 at every time point, indicating no alteration in these values (Online Resource 3). No DDI between pertuzumab and trastuzumab was expected in this study as pertuzumab and trastuzumab bind to distinct epitopes of HER2 simultaneously without steric hindrance [20]. The 90% CIs for the ratios were large because of variability in PK parameters and the relatively small sample size. Collectively, the results demonstrate that the exposure of trastuzumab was not affected by pertuzumab administration in the presence of chemotherapy, confirming what was shown previously in the small CLEOPATRA DDI sub-study [14]). Overall, the data suggested that pertuzumab did not alter the PK of trastuzumab, paclitaxel, or carboplatin, and that pertuzumab PK was not altered by concurrent trastuzumab administration. DDIs between pertuzumab and trastuzumab and between pertuzumab and docetaxel were previously assessed in a small sub-study of the CLEOPATRA trial [14]; however, the sample size was relatively small (*n* = 17 in the placebo arm and *n* = 20 in the pertuzumab arm) and the current analysis helps to confirm the previous results.

Efficacy (based on IDFS) and safety (based on any grade ≥ 3 AE and Grade ≥ 3 diarrhea) were compared between high and low pertuzumab exposure groups. Only four IDFS events occurred in 35 patients with PK data. Analyses of differences in exposure (AUC_ss_ and *C*_min,ss_) between patients with and without IDFS events did not indicate improved efficacy with higher pertuzumab exposure. While there was no indication of improved efficacy with higher pertuzumab exposure, the analysis is based on graphical assessment only and not a formal statistical test. Furthermore, the data were too limited to draw robust conclusions.

A difference in incidence of Grade ≥ 3 AEs was observed between treatment arms in the overall APHINITY study (64.2% in pertuzumab arm vs. 57.3% in placebo arm) [13]. This was also reflected in the APHINITY Global PK sub-study populations in which 27 events were observed in 35 patients treated with pertuzumab, compared with 25 events in 34 patients treated with placebo. Analyses of differences in pertuzumab exposure (below or above median *C*_max,ss_ and AUC_ss_) in patients with any grade ≥ 3 AEs showed a small difference between the exposure groups. Therefore, the available data did not suggest any exposure–safety relationship. It should, however, be noted that there was a limited number of patients in the pertuzumab-treated arm for robust statistical analyses.

A comparatively large difference in incidence of Grade ≥ 3 diarrhea was observed between the two treatment groups (20% in pertuzumab arm vs. 5.9% in placebo arm). The difference was similar to that seen in the overall APHINITY study (9.8% in pertuzumab arm vs. 3.7% in placebo arm). Analyses of differences in pertuzumab exposure (below or above median *C*_max,ss_ and AUC_ss_) did not indicate a higher incidence of Grade ≥ 3 diarrhea with higher exposure to pertuzumab.

In this exploratory APHINITY Global PK sub-study, the PK of pertuzumab in patients with HER2-positive EBC were in line with predictions from a previously developed and validated population PK model [10]. These analyses demonstrate that no dose adjustments are necessary for pertuzumab and trastuzumab when the two monoclonal antibodies are administered together with chemotherapy (anthracycline- or non-anthracycline-containing) in patients with EBC. It should, however, be noted that data were limited due to small sample sizes, and by the fact that only four patients had an IDFS event. Overall, the limited available data from this exploratory study suggest that no dose adjustments are needed for pertuzumab when administered in combination with trastuzumab and an EBC chemotherapy regimen.

## Electronic supplementary material

Below is the link to the electronic supplementary material.

Supplementary file1 (DOCX 33 kb)

Supplementary file2 Online Resource 2 Individual CWRES vs. population predictions. The open circles represent individual CWRES and the solid green line and shaded green area represent the Gaussian Loess smooth with 95% confidence interval. CWRES is conditional weighted residuals (PDF 978 kb)

Supplementary file3 (DOCX 30 kb)

Supplementary file4 Online Resource 4 Serum Cmax and AUClast of paclitaxel in Cycle 1 in presence of trastuzumab with or without pertuzumab. The closed circles represent paclitaxel in the treatment arm (pertuzumab, trastuzumab, and chemotherapy). The open circles represent paclitaxel in the control arm (placebo, trastuzumab, and chemotherapy). The solid green line represents arithmetic mean for each parameter and treatment arm. The shaded area is arithmetic mean ± 1 standard deviation. AUClast is area under the concentration–time curve over all concentration measurements, CI is confidence interval, Cmax is maximum serum concentration, SD is standard deviation (PDF 2037 kb)

Supplementary file5 Online Resource 5 Serum Cmax of 6-alpha-hydroxy paclitaxel in Cycle 1 in presence of trastuzumab with or without pertuzumab. The closed circles represent 6-alpha-hydroxy paclitaxel in the treatment arm (pertuzumab, trastuzumab, and chemotherapy). The open circles represent 6-alpha-hydroxy paclitaxel in the control arm (placebo, trastuzumab, and chemotherapy). The solid green line represents arithmetic mean for each treatment arm. The shaded area is arithmetic mean ± 1 standard deviation. CI is confidence interval, Cmax is maximum serum concentration, SD is standard deviation (PDF 1579 kb)

Supplementary file6 Online Resource 6 Serum Cmax and AUClast of carboplatin in cycle 1 in presence of trastuzumab with or without pertuzumab. The closed circles represent carboplatin in the treatment arm (pertuzumab, trastuzumab, and chemotherapy). The open circles represent carboplatin in the control arm (placebo, trastuzumab, and chemotherapy). The solid green line represents arithmetic mean for each parameter and treatment arm. The shaded area is arithmetic mean ± 1 standard deviation. AUClast is area under the concentration–time curve over all concentration measurements, CI is confidence interval, Cmax is maximum serum concentration, SD is standard deviation (PDF 2039 kb)
