# Supplementary material for: Pharmacokinetic and exploratory exposure–response analysis of pertuzumab in patients with operable HER2-positive early breast cancer in the APHINITY study
Source: Cancer Chemother Pharmacol. 2019 Apr 11;83(6):1147–58. doi: 10.1007/s00280-019-03826-1 (PMC6499763; doi:10.1007/s00280-019-03826-1)
Supplement: Supplementary file 1 — Supplementary file1 (DOCX 33 kb) [file 280_2019_3826_MOESM1_ESM.docx]

**Online Resource 1** Summary of demographics of patients in the APHINITY Study and Global PK sub-study

| Treatment Group |  | Placebo + trastuzumab + chemotherapy | Pertuzumab + trastuzumab + chemotherapy (no PK) | Global PK sub-study (pertuzumab) | Intention-to-treat |
| --- | --- | --- | --- | --- | --- |
|  |  | (*n* = 2404) | (*n* = 2362) | (*n* = 38) | (*n* = 4804) |
| Gender – no. of patients (%) | | | | | |
|  | Female | 2396 (99.7) | 2359 (99.9) | 38 (100.0) | 4793 (99.8) |
|  | Male | 8 (0.3) | 3 (0.1) | 0 (0.0) | 11 (0.2) |
| Age – year | | | | | |
|  | Mean (SD) | 51.4 (11) | 51.7 (11) | 51.8 (11) | 51.5 (11) |
|  | Median (range) | 51 (18–85) | 51 (22–86) | 51.5 (30–73) | 51 (18–86) |
|  | Missing (%) | 0 (0) | 0 (0) | 0 (0) | 0 (0) |
| LBW – kg | | | | | |
|  | Mean (SD) | 45.4 (5.2) | 45.4 (5.1) | 44.8 (4.1) | 45.4 (5.2) |
|  | Median (range) | 45.3  (17.6–73.0) | 45.2  (30.5-67.4) | 44.7  (34.8-56.8) | 45.2  (17.6–73.0) |
|  | Missing (%) | 8 (0.3) | 9 (0.4) | 0 (0) | 17 (0.4) |
| Race – no. of patients (%) | | | | | |
| American Indian or Alaska Native | | 56 (2.3) | 57 (2.4) | 0 (0.0) | 113 (2.4) |
| Asian | | 598 (24.9) | 587 (24.9) | 3 (7.9) | 1188 (24.7) |
| Black or African American | | 41 (1.7) | 32 (1.4) | 0 (0.0) | 73 (1.5) |
| Missing | | 2 (0.1) | 7 (0.3) | 0 (0.0) | 9 (0.2) |
| Native Hawaiian or other Pacific Islander | | 7 (0.3) | 3 (0.1) | 0 (0.0) | 10 (0.2) |
| Other | | 6 (0.2) | 6 (0.3) | 0 (0.0) | 12 (0.2) |
| White | | 1694 (70.5) | 1670 (70.7) | 35 (92.1) | 3399 (70.8) |
| Nodal status – no. of patients (%) | | | | | |
| 0 positive nodes and tumor <5 cm | | 84 (3.5) | 87 (3.7) | 3 (7.9) | 174 (3.6) |
| 0 positive nodes and tumor >1 cm | | 818 (34.0) | 792 (33.5) | 15 (39.5) | 1625 (33.8) |
| 1–3 positive nodes | | 900 (37.4) | 895 (37.9) | 12 (31.6) | 1807 (37.6) |
| >4 positive nodes | | 602 (25.0) | 588 (24.9) | 8 (21.1) | 1198 (24.9) |
| Hormone receptor status – no. of patients (%) | | | | | |
| Negative | | 814 (33.9) | 805 (34.1) | 13 (34.2) | 1632 (34.0) |
| Positive | | 1590 (66.1) | 1557 (65.9) | 25 (65.8) | 3172 (66.0) |

*PK* is pharmacokinetic, *SD* is standard deviation
