# Supplementary figures and images for: Pharmacokinetic and exploratory exposure–response analysis of pertuzumab in patients with operable HER2-positive early breast cancer in the APHINITY study
Source: Cancer Chemother Pharmacol. 2019 Apr 11;83(6):1147–58. doi: 10.1007/s00280-019-03826-1 (PMC6499763; doi:10.1007/s00280-019-03826-1)

Conditional weighted residual

4  
2  
0  
-2  
-4

100

Population predictions ( $\mu\text{g/mL}$ )

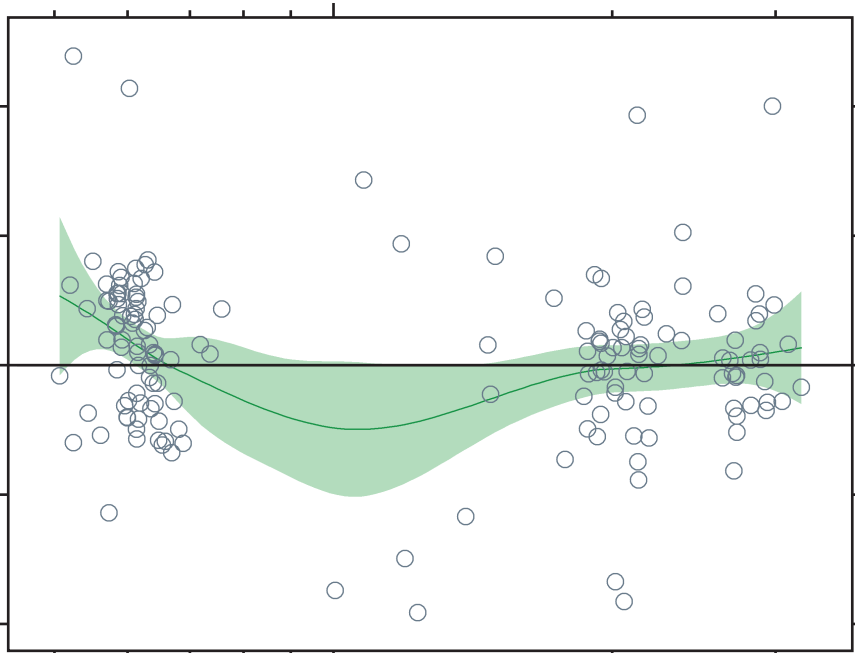

Supplement: Supplementary file 2 — Supplementary file2 Online Resource 2 Individual CWRES vs. population predictions. The open circles represent individual CWRES and the solid green line and shaded green area represent the Gaussian Loess smooth with 95% confidence interval. CWRES is conditional weighted residuals (PDF 978 kb) [file 280_2019_3826_MOESM2_ESM.pdf]
