# Supplementary material for: Pharmacokinetic and exploratory exposure–response analysis of pertuzumab in patients with operable HER2-positive early breast cancer in the APHINITY study
Source: Cancer Chemother Pharmacol. 2019 Apr 11;83(6):1147–58. doi: 10.1007/s00280-019-03826-1 (PMC6499763; doi:10.1007/s00280-019-03826-1)
Supplement: Supplementary file 3 — Supplementary file3 (DOCX 30 kb) [file 280_2019_3826_MOESM3_ESM.docx]

**Online Resource 3** Summary of serum C_min_ and C_max_ of trastuzumab in the presence of chemotherapy with or without pertuzumab

| Timepoint | Pertuzumab + trastuzumab + chemotherapy,  mean (± SD) | n | Placebo + trastuzumab + chemotherapy,  mean (± SD) | n | Geometric mean ratio  (90 % CI) |
| --- | --- | --- | --- | --- | --- |
| Cycle 1 C_max_ | 179.1 (± 69.1) | 34 | 189.9 (± 51.6) | 33 | 0.873 (0.71–1.07) |
| Cycle 1 C_min_ | 30.8 (± 11.3) | 31 | 34.1 (± 11.4) | 31 | 0.874 (0.75–1.03) |
| Cycle 10 C_max_ | 226.3 (± 87.4) | 32 | 224.5 (± 70.7) | 27 | 0.978 (0.80–1.2) |
| Cycle 10 C_min_ | 67.0 (± 38.5) | 32 | 68.4 (± 23.0) | 26 | 0.878 (0.71–1.09) |
| Cycle 15 C_max_ | 195.1 (± 88.6) | 24 | 223.6 (± 73.5) | 21 | 0.765 (0.61–0.968) |
| Cycle 15 C_min_ | 75.7 (± 44.6) | 26 | 71.0 (± 30.4) | 22 | 0.967 (0.74–1.27) |

Arithmetic means. Serum concentrations in μg/mL.

*CI* is confidence interval, *C_max_* is maximum serum concentration, *C_min_* is minimum serum concentration, *SD* is standard deviation
