# Supplementary material for: Pharmacokinetic and exploratory exposure–response analysis of pertuzumab in patients with operable HER2-positive early breast cancer in the APHINITY study
Source: Cancer Chemother Pharmacol. 2019 Apr 11;83(6):1147–58. doi: 10.1007/s00280-019-03826-1 (PMC6499763; doi:10.1007/s00280-019-03826-1)
Supplement: Supplementary file 5 — Supplementary file5 Online Resource 5 Serum Cmax of 6-alpha-hydroxy paclitaxel in Cycle 1 in presence of trastuzumab with or without pertuzumab. The closed circles represent 6-alpha-hydroxy paclitaxel in the treatment arm (pertuzumab, trastuzumab, and chemotherapy). The open circles represent 6-alpha-hydroxy paclitaxel in the control arm (placebo, trastuzumab, and chemotherapy). The solid green line represents arithmetic mean for each treatment arm. The shaded area is arithmetic mean ± 1 standard deviation. CI is confidence interval, Cmax is maximum serum concentration, SD is standard deviation (PDF 1579 kb) [file 280_2019_3826_MOESM5_ESM.pdf]

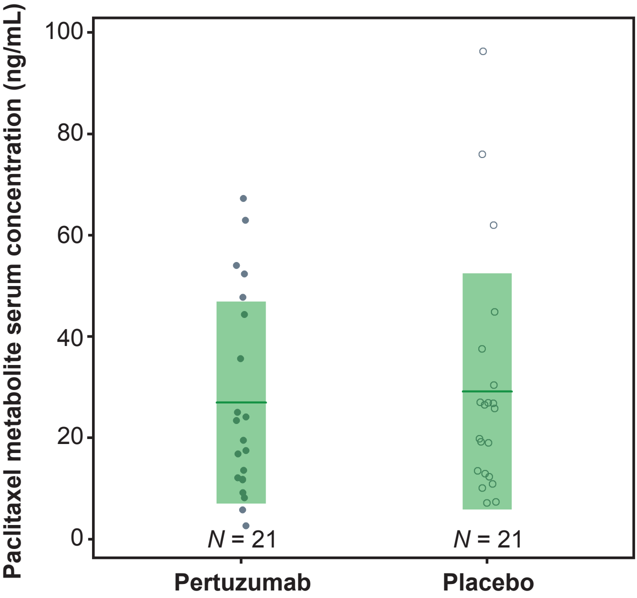

	Pertuzumab + trastuzumab + chemotherapy, mean (± SD)	Placebo + trastuzumab + chemotherapy, mean (± SD)	Geometric mean ratio (90% CI)
n	21	21	–
C_{max}	27.0 (± 19.9)	29.2 (± 23.3)	0.882 (0.587–1.33)

Arithmetic means. Serum C_{max} in μg/mL.
