# Supplementary material for: Pharmacokinetic and exploratory exposure–response analysis of pertuzumab in patients with operable HER2-positive early breast cancer in the APHINITY study
Source: Cancer Chemother Pharmacol. 2019 Apr 11;83(6):1147–58. doi: 10.1007/s00280-019-03826-1 (PMC6499763; doi:10.1007/s00280-019-03826-1)
Supplement: Supplementary file 6 — Supplementary file6 Online Resource 6 Serum Cmax and AUClast of carboplatin in cycle 1 in presence of trastuzumab with or without pertuzumab. The closed circles represent carboplatin in the treatment arm (pertuzumab, trastuzumab, and chemotherapy). The open circles represent carboplatin in the control arm (placebo, trastuzumab, and chemotherapy). The solid green line represents arithmetic mean for each parameter and treatment arm. The shaded area is arithmetic mean ± 1 standard deviation. AUClast is area under the concentration–time curve over all concentration measurements, CI is confidence interval, Cmax is maximum serum concentration, SD is standard deviation (PDF 2039 kb) [file 280_2019_3826_MOESM6_ESM.pdf]

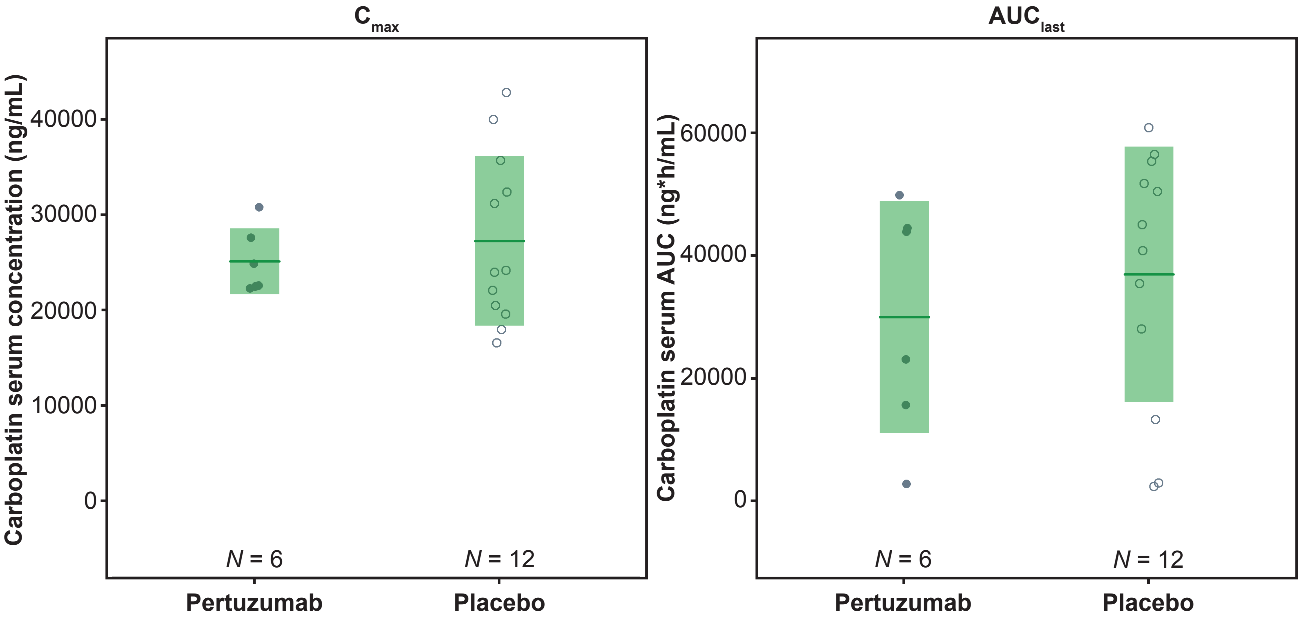

	Pertuzumab + trastuzumab + chemotherapy, mean (± SD)	Placebo + trastuzumab + chemotherapy, mean (± SD)	Geometric mean ratio (90% CI)
n	6	12	–
C_{max}	25116.7 (± 3453.4)	27258.3 (± 8879.7)	0.959 (0.795–1.16)
AUC_{last}	29986.2 (± 18914.6)	36940.5 (± 20804.7)	0.833 (0.307–2.26)

Arithmetic means. Serum C_{max} in μg/mL. Serum AUC_{last} in ng·h/mL.
